# A microarray-based method for the parallel analysis of genotypes and expression profiles of wood-forming tissues in *Eucalyptus grandis*

**DOI:** 10.1186/1472-6750-9-51

**Published:** 2009-05-27

**Authors:** Eugenia Barros, Carol-Ann van Staden, Sabine Lezar

**Affiliations:** 1 CSIR – Biosciences, Meiring Naude Road, Brummeria, Pretoria, 0001, South Africa

## Abstract

**Background:**

Fast-growing *Eucalyptus grandis *trees are one of the most efficient producers of wood in South Africa. The most serious problem affecting the quality and yield of solid wood products is the occurrence of end splitting in logs. Selection of *E. grandis *planting stock that exhibit preferred wood qualities is thus a priority of the South African forestry industry. We used microarray-based DNA-amplified fragment length polymorphism (AFLP) analysis in combination with expression profiling to develop fingerprints and profile gene expression of wood-forming tissue of seven different *E. grandis *trees.

**Results:**

A 1578-probe cDNA microarray was constructed by arraying 768 cDNA-AFLP clones and 810 cDNA library clones from seven individual *E. grandis *trees onto silanised slides. The results revealed that 32% of the spotted fragments showed distinct expression patterns (with a fold change of at least 1.4 or -1.4 and a p value of 0.01) could be grouped into clusters representing co-expressed genes. Evaluation of the binary distribution of cDNA-AFLP fragments on the array showed that the individual genotypes could be discriminated.

**Conclusion:**

A simple, yet general method was developed for genotyping and expression profiling of wood-forming tissue of *E. grandis *trees differing in their splitting characteristics and in their lignin contents. Evaluation of gene expression profiles and the binary distribution of cDNA-AFLP fragments on the chip suggest that the prototype chip developed could be useful for transcript profiling and for the identification of Eucalyptus trees with preferred wood quality traits in commercial breeding programmes.

## Background

Eucalyptus tree species are an extremely important source of hardwood for forest industries worldwide. It is the most widely planted hardwood species in the temperate, sub-tropical and tropical zones. In South Africa, about 1.26 million ha are *Eucalyptus *plantations which accounts for 37.7% of total forest plantations [1]. *Eucalyptus grandis *are the most commonly used trees in forest plantations. The most serious problem affecting wood quality and product yield of South African *Eucalyptus *trees is the high level of growth stress that develops as the trees grow, manifesting itself in severe splitting when the trees are felled and cut into logs [2]. Molecular markers linked to wood splitting in *E. grandis *were developed by Barros *et al*. [3] and were successfully used in the selection of non-splitting clones as part of a marker-assisted breeding programme. However, no genes linked to the differential response of *E. grandis *trees to wood end splitting and to growth stress were identified. Growth stress arises from the deposition of lignin within the secondary walls during maturation of fibrous cells, which includes the biosynthesis of polysaccharides and cell wall proteins [4,5]. The genes and genetic mechanisms that underlie growth stress are of particular interest in *E. grandis *due to the potential of identifying trees with desirable wood properties.

A variety of molecular techniques are available to identify differentially expressed genes. cDNA-AFLP has been successfully used in the identification of a wide range of candidate genes including genes with possible roles in plant defense response [6], in fruit ripening processes [7] and in cell wall biosynthesis in Eucalyptus [8]. Recently, expressed sequence tag (EST) sequencing proved to be an efficient approach to identify gene types and novel genes during wood formation. Using this technique Allona *et al*. [9] found a significant representation of cellulose, lignin and other cell-wall biosynthesis genes and a comparable percentage of ESTs. Pavy *et al*. [10] identified a total of 260 differentially expressed gene sequences and the gene encoding the Smad4 interacting factor by statistical analysis of ESTs belonging to the TIGR Pinus Gene Index. In a similar experiment, Ramussen-Poblete *et al*. [11] described transcription factor families such as the AUX/IAA (auxin/indole-3-acetic acid) family, MYB 9 and HD containing domains (zinc finger proteins and homeodomain-leucine zipper) that regulate genes participating in xylem development and secondary cell wall formation (lignin and cellulose biosynthesis) [12,13].

The accumulation of large EST libraries have allowed the construction of high-throughput cDNA microarray chips [5], which could be used to study gene expression in tree species such as Eucalyptus [14-16], pine [17,18], poplar [19] and aspen [20]. Demura *et al*. [21] used cDNA microarrays to identify clusters of *Zinnia elegans *genes that punctuate the major morphological and biochemical events of the transdifferentiation of tracheary elements [8]. Further evidence of the identity of major genes involved in wood formation has been gained in a separate study by Foucart *et al*. [22] who established a portfolio of Eucalyptus xylem genes. This technique can also be used to assay DNA sequence variation in different phenotypes reducing the genotyping effort as well as producing quantitative raw data that can then be converted into discrete genotypes and has been used in several studies [23-26].

A strategy that combines high-throughput microarray expression profiling with genotyping offers the opportunity to explore gene expression of a tree that has not been completely characterized at the molecular level. The aim of the study was to develop a prototype microarray chip from differentiating xylem tissue of *E. grandis *trees differing in their splitting characteristics and in their lignin contents. cDNA-AFLP and cDNA microarray analysis was used to identify individual *E. grandis *trees exhibiting preferred wood qualities and to identify differential expressed genes underlying different aspects of wood development that could help elucidate wood splitting. This study also evaluates the potential of combining expression analysis with fingerprinting analysis for the early detection of *E. grandis *trees that are prone to severe splitting. Trees identified as being prone to splitting could be excluded from breeding populations and add value to plantation forestry.

## Results

### RNA and cDNA quality

The RNA extracted from wood-forming tissue of the seven *E. grandis *trees was found to be of high quality and the absence of contaminating genomic DNA was confirmed for all cDNA samples (results not shown). The amplification of a region of the CAD2 genes from cDNA yielded the expected 410 bp mRNA-derived amplicon, which was clearly distinguishable from the 700 bp genomic DNA-derived, intron-containing fragments (results not shown).

### Assembly of clones from cDNA library and cDNA-AFLP

cDNA libraries were constructed from RNA extracted from the seven *E. grandis *trees and a total of 810 cDNA clones were arrayed onto a microarray slide to be used for transcript profiling of the trees. The cDNA-AFLP clones were also generated from the seven trees and the selective amplification using the single +3 *Mse*/+2*Pst *primer combination showed high variable expression levels among the trees. Amplified fragments ranged in sizes from 100 bp to over 700 bp when visualized on polyacrylamide gels. These fragments were cloned and spotted onto the same microarray slide. In total 768 cDNA-AFLP clones were spotted to be used for the identification, fingerprinting and expression profiling of *E. grandis *trees.

### Analysis of the combined array

The combined microarray containing a total of 1578 clones was hybridized with cDNA from the seven *E. grandis *trees. Hybridization profiles showed that 193 (12.6%) cDNA-AFLP clones and 206 (13.4%) cDNA library clones were differentially expressed. This revealed that both approaches generated a similar amount of differentially expressed clones suggesting that both techniques are equally useful for expression profiling.

### General expression patterns of the combined array

**Figure 1:**
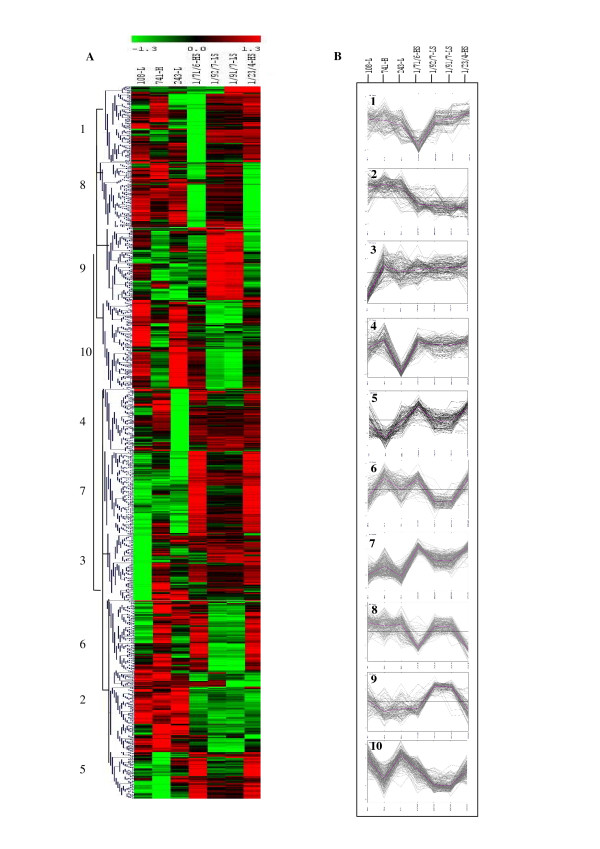
**Hierarchical clustering of expression patterns**. **A**. Hierarchical clustering of 1578 differentially expressed tissue profiles from the combined cDNA array. Relative expression levels (median centered and standardized values) are represented by a continuum with green signifying relative low expression of the ESTs, black indicating moderate expression (relative up-regulation) in the respective tissues, and red indicating high expression. The rows correspond to the quantified ESTs and columns to the respective *E. grandis *trees. LS: low splitting, HS: high splitting, H: high lignin, and L: low lignin, all collected from the lower-half of the stem. The transcripts have been divided into 10 expression pattern clusters as indicated by the numbers 1–10. **B**. Graphs of expression pattern clusters. Vertical axes in B represent standard deviation from the median expression level of each gene. The location of each cluster is indicated in A.

A 1578-probe prototype cDNA microarray was constructed by arraying selective amplifications (*Mse*3/*Pst*4) of 768 cDNA-AFLP fragments and 810 cDNA library clones from seven individual *Eucalyptus *trees onto silanized glass slides. The cDNA profiles were clustered according to their expression patterns using Pearson's correlation in the Cluster program of Eisen *et al *[27] and are shown in Fig. 1A. Based on the clustering, ten different groups of co-expressed genes could be annotated (Fig. 1B). Clusters 3 and 4 contain genes that were up-regulated in the high lignin and the two high splitting trees. Most transcripts represented in these clusters are involved in cell wall biogenesis and include genes such as glucuronic acid decarboxylase 3 (UXS3), xyloglucan endotransglycosylate (XET) and caffeoyl-CoA 3-O-methyltransferase (CCoAOMT). Cluster 10 represents genes that were up-regulated in the high lignin and in the two high splitting trees. Most transcripts belonging to this cluster are associated with stress/defense. One transcript belonging to cluster 2, the putative zinc finger protein, was up-regulated in one low lignin and in the two low splitting trees. This gene is also part of the stress group. In general it was observed that stress-related genes were mostly up-regulated in the high lignin and high splitting trees. A similar pattern was also observed for the transcripts responsible for the higher lignin content which were up-regulated in both the high splitting trees and high lignin trees.

The combined microarray was further analyzed by determining the statistical significance of changes in transcript abundance using the method described by Wolfinger *et al*. [28]. Eighty clones were found to be distinctly differentially expressed with a fold change of at least 1.4 or -1.4 and a p value of 0.01. These clones representing different gene clusters were isolated and functionally classified based on the MIPS standard [29]. This revealed that 10% of the expressed sequence tags (ESTs) were involved in cell wall biogenesis, 4% in cell growth, 4% in protein metabolism, 2% in transcription, 1% in energy, 5% in metabolism, 4% in signal transduction and 2% in stress (Additional file 1). A high proportion of the ESTs (67%) were classified as either having unknown function (21%) or as not producing any hits (46%). At least ten cDNA clones of each distinct group were sequenced to get precise information on their potential functions. Fragments sequenced fitted into the broad classification of similarity-inferred EST identities based on BLASTX results. The putative functions of the fragments sequenced are listed in Table 1.

**Table 1:** Differentially expressed transcripts of seven *E. grandis *trees and their putative functions

				**Fold change^a^**						
				
**Functional category**	**NCBI Accession number**	**Cluster**	**Putative identity**	**1**	**2**	**3**	**4**	**5**	**6**	**7**
**Energy**										
Glycolysis	AAM94349.1	-	Pyruvate kinase	-1.0	-1.0	-1.4	1.2	1.0	1.0	1.4
Other	AY674766.1	7	*Sabia swinhoei *NADH dehydrogenase subunit 1 gene, exons 2, 3	-1.2	-1.4	-1.4	1.2	1.0	1.0	1.2
**Cell growth**										
Cell growth	AJ841794.1	5	Populus × canadensis mRNA for putative histidine-containing phosphotransfer protein 2 (hpt2 gene), cultivar Dorskamp	1.2	-1.0	-1.6	1.0	1.0	1.0	1.2
**Transcription**										
Posttranscriptional	AAZ32862.1	2	Putative splicing factor Prp8 (*Medicago sativa*)	1.0	-1.2	-1.4	-1.4	1.2	1.4	-1.6
**Protein synthesis**										
Protein synthesis	023627	1	Putative glycyl tRNA synthetase, *Arabidopsis thaliana*	1.0	1.0	1.0	-1.6	1.2	1.0	1.2
**Metabolism**										
Metabolism	ABE86334.1	1	D-isomer specific 2-hydroxyacid dehydrogenase, catalytic region (*Medicago trunculata*)	1.0	1.0	1.0	-1.4	1.0	1.2	1.0
Metabolism	AAT12488.1	1	Copper chaperone (Populus alba × Populus tremula var. glandulosa)	1.2	1.0	1.0	-1.6	1.2	1.0	1.0
Metabolism	AY686850.1	6	Streblus smithii voucher G. Weiblen 1172 (MIN) 26S ribosomal RNA, partial sequence	1.2	1.2	1.0	-1.4	1.0	1.2	-1.2
Protein phosphorylation	AY227028.1	6	*Chlamydomonas reinhardtii *GMP synthetase gene	1.0	1.0	1.2	-1.6	1.0	1.2	-1.2
**Cell biogenesis**										
Cell wall	CT981136.1	4	Partial cDNA sequence of caffeoyl-CoA 3-O-methyltransferase from *Eucalyptus gunnii*	-1.6	1.6	1.2	-1.4	1.0	-1.4	-1.0
Unknown	CAB80346.1	4	Ubiquitin – protein ligase-like protein (*Arabidopsis thaliana*)	1.4	1.6	1.0	1.2	1.0	-1.4	-1.0
Cell wall	AJ627732	3	Putative xyloglucan endo-transglycosylase (XET)	-1.2	1.2	-1.4	1.4	-1.0	-1.0	1.4
Cell wall	AY922315.1	3	*Populus tomentosa *UDP-glucuronic acid decarboxylase 3 (UXS3)	-1.0	1.0	-1.4	1.2	-1.2	-1.0	1.4
Cell wall	Zp00567351.1	5	Protein-L-isoaspartate O-methyltransferase	1.4	1.2	1.4	1.0	-1.4	-1.4	1.0
Cell wall	Q59296	5	Catalase	1.4	1.0	1.4	1.2	-1.4	-1.4	1.2
**Signal transduction**										
Kinase	Q42806	9	Pyruvate kinase, cytosolic isozyme (PK)	-1.0	-1.0	-1.0	1.0	1.0	-1.2	-1.4
Hormone	AJ508907.1	9	mRNA for allototropin (at gene)	-1.0	-1.0	-1.2	1.2	1.2	-1.0	-1.4
**Stress**										
Transcription	ABA98970.2	2	Putative Zinc-finger protein	1.2	-1.4	-1.0	-1.4	1.2	1.2	-1.6
Defense	NP 172668.1	10	LRX1 Leucine-Rich Repeat/EXTENSIN 1	-1.0	1.4	-1.0	1.0	-1.0	-1.0	-1.0
Defense	AAN62344	10	CTV.15	-1.2	1.2	-1.2	1.4	-1.0	-1.2	-1.0
Defense	ABA91873.1	10	Lipase family protein	-1.2	1.2	-1.4	1.2	-1.0	-1.2	1.2
Defense	Q8CCP0	10	Antigen 1	-1.0	1.2	1.2	1.0	-1.2	-1.4	1.4

^a^Numbers indicate individuals 1 (108-L), 2 (741-H), 3 (243-L), 4 (1/71/6-HS), 5 (1/92/7-LS), 6 (1/91/7-LS) and 7 (1/23/4-HS)

The majority of ESTs were up-regulated in the low splitting trees and seem to be involved in metabolism and transcription. A D-isomer specific 2-hydroxyacid dehydrogenase, which has oxidoreductase activity, a 26S ribosomal RNA, a putative glycyl tRNA synthetase and a splicing factor Prp8 were identified in these groups. Most of the transcripts belong to the functional category cell wall biogenesis and include transcripts such as glucuronic acid decarboxylase 3 (UXS3), xyloglucan endotransglycosylate (XET) and caffeoyl-CoA 3-O-methyltransferase (CCoAOMT). All three transcripts are known to play a fundamental role in regulating cell wall architecture and mechanical strength [30]. A second group of candidate transcripts that were up-regulated in the high lignin and high splitting trees were associated with stress/defense-related functions. This group contains transcripts such as a leucine-rich repeat, a lipase family protein and an antigen. Only a putative zinc finger protein in this group was up-regulated in one low lignin and in the two low splitting trees. All the transcripts associated with stress/defense are known to be strongly expressed in response to stress during secondary cell wall synthesis [31].

### Quantitative expression analysis of cDNA-AFLP clones

Quantitative expression data for cDNA-AFLP clones was obtained by assaying the presence or absence of microarray markers using the hybridization patterns of the 768 *E. grandis *clones among trees. Generally, fragments on the array ranged in size from 100 bp to over 700 bp. Of 768 clones spotted onto the slide 133 (17.3%) were found to be polymorphic among the trees. The analysis was limited to only those spots for which clear threshold values (difference of 0.5 in relative intensity between two intensity classes) could be assigned. Spots with clear threshold values could be easily converted to binary scores (see Additional file 2). A unique microarray pattern was obtained for each *Eucalyptus *tree. Hybridisation profiles resulting from individual trees were 96% identical to those obtained in replicate, by reverse labelling reactions.

### qRT-PCR verification

The combined cDNA array analysis was representative of differentially expressed transcripts from seven *E. grandis *trees. Real-time PCR was performed on five ESTs representing different expression clusters to verify the accuracy of cDNA microarray quantification. The ESTs chosen included UDP-glucuronic acid decarboxylase 3 (UXS3), a histidine-containing phosphotransfer protein 2 (hpt2), D-isomer specific 2-hydroxyacid dehydrogenase, caffeoyl-CoA 3-O-methyltransferase (CCoAOMT) and protein-L-isoaspartate O-methyltransferase. Microarray analysis suggested that ribosomal RNA was expressed constitutively and was, therefore, used as a control for normalization of the real-time PCR data. Analysis of results from both microarray data and qRT-PCR showed that the trends and patterns are consistent between the two different methods (Table 2). The higher fold values of transcripts detected by qRT-PCR were expected.

**Table 2:** Verification of array results

			**Fold change**	
			
**NCBI Accession number**	**Putative identity**	**Tree**	**Array**	**qRT-PCR**
AY922315.1	*Populus tomentosa *UDP-glucuronic acid decarboxylase 3 (UXS3)	108-L	-1.0	-1.35 ± 0.09
		741-H	1.0	1.27 ± 0.01
		243-L	-1.4	-2.04 ± 0.18
		1/71/6-HS	1.2	1.41 ± 0.01
		1/92/7-LS	-1.2	-1.70 ± 0.05
		1/91/7-LS	-1.0	-1.80 ± 0.01
		1/23/4-HS	1.4	2.02 ± 0.17
CT981136.1	Partial cDNA sequence of caffeoyl-CoA 3-O-methyltransferase (CCoAOMT) from *Eucalyptus gunnii*	108-L	-1.6	-3.95 ± 0.03
		741-H	1.6	3.45 ± 0.18
		243-L	-1.2	-3.14 ± 0.01
		1/71/6-HS	1.4	3.45 ± 0.01
		1/92/7-LS	1.0	2.24 ± 0.10
		1/91/7-LS	-1.4	-3.86 ± 0.16
		1/23/4-HS	-1.0	-2.30 ± 0.03
Zp00567351.1	Protein-L-isoaspartate O-methyltransferase	108-L	1.4	1.53 ± 0.04
		741-H	1.2	1.57 ± 0.06
		243-L	1.4	1.67 ± 0.06
		1/71/6-HS	1.0	1.83 ± 0.06
		1/92/7-LS	-1.4	-1.73 ± 0.05
		1/91/7-LS	-1.4	-1.81 ± 0.02
		1/23/4-HS	1.0	1.68 ± 0.11
AJ841794.1	Populus × canadensis mRNA for putative histidine-containing phosphotransfer protein 2 (hpt2 gene), cultivar Dorskamp	108-L	1.2	1.38 ± 0.04
		741-H	-1.0	-1.36 ± 0.04
		243-L	-1.6	-2.38 ± 0.01
		1/71/6-HS	1.0	1.41 ± 0.01
		1/92/7-LS	1.0	1.51 ± 0.01
		1/91/7-LS	1.0	1.76 ± 0.05
		1/23/4-HS	1.2	1.84 ± 0.06
ABE86334.1	D-isomer specific 2-hydroxyacid dehydrogenase, catalytic region (*Medicago trunculata*)	108-L	1.0	1.18 ± 0.04
		741-H	1.0	1.38 ± 0.15
		243-L	1.0	1.21 ± 0.01
		1/71/6-HS	-1.4	-1.40 ± 0.02
		1/92/7-LS	1.0	1.38 ± 0.08
		1/91/7-LS	1.2	1.62 ± 0.03
		1/23/4-HS	1.0	1.44 ± 0.01

Confirmation of the expression patterns of five clusters by relative quantification of transcript abundance using qPCR. Fold change (± standard deviation) of a subset of *E. grandis *transcripts chosen for their functional relevance.

## Discussion

Identification of superior *E. grandis *trees not prone to growth stress is essential for maximising the effectiveness of plantations adding value to the forestry industry. The genes and genetic mechanisms that underlie growth stress are of particular interest in *E. grandis*, due to the potential of selecting trees prone to severe splitting that could be excluded from breeding populations. In this study, a 1578-probe cDNA microarray was developed for both genotyping and expression profiling using seven different *E. grandis *trees. The combined microarray offers an opportunity to discriminate between individual trees as well as analyze transcript abundance, variability and the usefulness of the chip for fingerprinting.

For the transcript profiling and genotyping, a 1578-probe prototype cDNA microarray was constructed by arraying 768 cDNA-AFLP fragments and 810 cDNA library clones from seven *Eucalyptus *trees onto silanized glass slides. This provided an overview of transcript abundance, variability and the usefulness of the chip for fingerprinting transcripts. Analysis of the cDNA clones suggested that a significant proportion of genes expressed in the wood forming tissues of *Eucalyptus *are strongly up- or down-regulated. The high variability in gene expression patterns demonstrates that the sampling strategy used was successful in separating differentiating xylem tissue from the seven *E. grandis *trees and shows the extent to which the tissues of different tree phenotypes differ in function, biochemistry and morphology.

Clustering of expression profiles allowed the identification of distinct groups of co-expressed genes. These distinct groups may contain genes that are involved in the main metabolic or developmental processes occurring during tissue differentiation. A total of 80 differentially expressed transcripts representing different gene clusters were isolated and characterized. Ten percent of differentially abundant transcripts were identified as having roles in cell wall biogenesis. Two of them, glucuronic acid decarboxylase 3 (UXS3) and xyloglucan endotransglycosylate (XET), were found to be up-regulated in the high lignin tree and in the two high splitting trees. The increased expression of the UXS3 gene in the high lignin tree was expected as this gene was shown to be a precursor of xylan production in Arabidopsis [32,33]. Xylan is a component of hemicellulose and an increase in xylan will result in increased lignin. The up-regulation of the UXS3 gene in the two high splitting trees could be the result of the involvement of this gene in cell wall biosynthesis. This gene is responsible for the organization of cellulose, hemicellulose and lignin in cell walls, and therefore determining the mechanical strength of the cell wall. The XET gene which is similar to the Arabidopsis XET protein XTH9 [34] and to poplar XET gene (PttXET16A) [35] was found to play a fundamental role in the construction and modification of cell wall architecture. Nishikubo *et al*. [36] observed that the XET gene is involved in the repair of xyloglucan cross-linkages, creating and reinforcing the connections between the primary cell wall and the secondary cell wall layers. Since the XET gene was found to be up-regulated in the two high-splitting trees its role in wood splitting and in growth stress could be speculated. Growth stress originates in the cambial region of the stem during the maturation of the cells where the contraction of the cellulose molecule during lignin deposition is a contributing factor to the stress [37]. High splitting trees are thought to have elevated levels of growth stress and thus the higher expression of the XET gene. This could confirm the greater activity of this gene in expanding the cell wall during secondary cell wall thickening. The growth stress in the trees is in equilibrium but as soon as it is cut, and this state of balance is modified, log deformations and splits occur. Equally interesting is the transcript profiling pattern of genes encoding important enzymes in lignin biosynthesis, such as caffeoyl-CoA 3-O-methyltransferase (CCoAOMT). This gene is a key transcript directly associated with lignin biosynthesis [38] and has been characterized in tobacco [39] and poplar [40]. Paux *et al*. [41] reported that the CCoAOMT gene was up-regulated in *Eucalyptus gunni *xylem and this gene was shown to be involved in cell wall formation. The up-regulation of the CCoAOMT gene in the high lignin tree was expected as this gene is involved in lignin biosynthesis. The up-regulation of CCoAOMT in the high splitting trees suggests that this gene responds to signaling mechanisms and triggers a stress-related compensatory deposition of lignin.

The second largest group of candidate transcripts identified was associated with stress/defence-related functions. Most the transcripts in this group were up-regulated in the high lignin and in the two high splitting trees suggesting that the cells in the xylem layer could play a role in protecting the cambium under stress conditions. Only one transcript associated with stress, a putative zinc finger protein, was up-regulated in the two low splitting trees and in the low lignin tree. Zinc finger proteins have been speculated to interact with cellulose [42] and to be strongly expressed in response to gravitational stress during secondary cell wall synthesis [15,16,31]. The up-regulation of the gene coding for this protein in the low lignin and low splitting trees could not be explained at this stage.

Several studies in forest trees have reported high proportions of sequences lacking similarity to any known proteins [9,43-46]. In this study, similar results were obtained. Many transcripts showed no significant homologies to publicly available sequences. A high proportion of cDNA clones (67%) were classified as transcripts lacking similarity to any known sequences (21%) or as transcripts not producing any hits (46%). The genes of unknown function are most probably transcripts that are highly and specifically expressed in wood-forming tissues. These differentially expressed genes are a source of novel genes whose function should be characterized in future studies to determine their role in secondary xylem formation and, represent an important source of candidate genes to improve the quality of wood in *E. grandis*.

Another important aim of this study was the development of a combined microarray for the characterization and genotyping of *E. grandis *trees for future breeding programmes. The observed high variability in gene expression patterns among the seven individual trees representing the four phenotypes provided a starting point for the clustering of the 768 cDNA-AFLP clones. In this context, direct comparison of signal intensity profiles suggest that the cDNA chip developed will allow the genome-wide fingerprinting of the seven *E. grandis *genomes since a unique microarray pattern was obtained for each individual tree. Some of the genes preferentially and/or specifically expressed in Eucalyptus cambium were shown to exhibit a distinctive expression pattern, which could be related to the bimodal distribution of the expression patterns.

## Conclusion

A new microarray prototype was constructed that combined expression profiling and genotyping of *E. grandis *trees. This provides a tool for the identification and characterization of trees with superior qualities in breeding programmes. Furthermore expression level analysis gave a perspective of the types of genes active in wood-forming tissues while genotyping allowed the identification of individual trees. The genetic markers identified in this study in the form of genes that are either up- or down-regulated in the four different phenotypes could be used to develop gene-specific markers. The long-term objective of this study is to use the combined microarray for the identification of individual trees prone to splitting and for the identification of novel genes targeted to specific pathways. Novel genes for which no function has yet been assigned may hold the key towards a better understanding of the developmental processes and biochemical pathways that underlie wood formation and could be the source of candidate genes to improve the quality of wood in *E. grandis*.

## Methods

### Plant materials and tissue harvesting

Differentiating xylem tissue samples were collected from each of seven 4-year old coppice re-growth *E. grandis *trees that belonged to two unrelated, open pollinated trials, called the 'Florida' and the 'Frankfort' trials. The 'Florida' trial was established from seed imported from Florida, USA and the 'Frankfort' trial was established from South African plantation trees. The *E. grandis *trees were originally planted in 1979 and felled in 1999. All trees were characterized for their splitting qualities and lignin content as described by Turner [47]. Seven trees that best corresponded to the two selected traits were used in this study and are shown in Additional file 3 along with the trait for which they were selected. Two low lignin and one high lignin tree were selected from the 'Florida' trial and two high splitting and two low splitting trees were selected from the 'Frankfort' trial. For total RNA extraction a section of the stem of the coppice was progressively debarked and the exposed xylogenic tissue was scrapped, immediately frozen and stored at -80°C.

### Total RNA extraction, quality control and cDNA synthesis

Total RNA was isolated from xylem tissue of seven *E. grandis *trees (741-H (high lignin), 108-L (low lignin), 243-L (low lignin), 1/23/4-HS (high splitting), 1/71/6-HS (high splitting), 1/91/7-LS (low splitting) and 1/92/7-LS (low splitting)) as described by Chang *et al*. [48]. The total RNA was DNAse (Roche Diagnostics GmbH) treated and using an Oligotex^® ^mRNA Mini Kit (QIAGEN, Valencia, CA). RNA concentration was estimated using a ND-1000 Spectrophotometer (NanoDrop USA, Wilmington, DE) and integrity was evaluated on an agarose gel stained with ethidium bromide. Double-stranded cDNA was synthesized from purified RNA using the cDNA Synthesis System (Roche Diagnostics, Mannheim, Germany) according to manufacturer's protocol. cDNA was subsequently column-purified using the QIAquick PCR Purification Kit (QIAGEN). The purified cDNA were assayed for genomic DNA contamination by PCR using four separate intron-extron boundary spanning primer pairs: CCR.34-F1 (ACGTTGTGGTGGACGAGTC) and CCR.34-R1 (ACGTATGCCTGGACCGAGT) specific for the *E. globulus *cinnamoyl CoA reductase (CCR) gene; CCR1.23-F1 (CTTGTTGGAGCGACCTCGAA) and CCR1.23-R1 (ACGTACGCCTGGACCGAGTT) specific for the *E. gunnii *CCR1 gene; CAD.34-F1 (CTTGCAATTCGGACCAGGA) and CAD.34-R1 (GCTCCAATGCCTCCGTTCT) specific for *E. saligna *cinnamyl alcohol dehydrogenase gene; CAD.45-F1 (TCGCGATGCTTACCTAGTGAG) and CAD.45-R1 (CACGACGAACCTGTACCTGAC) specific for the *E. gunnii *cinnamyl alcohol dehydrogenase gene (CAD2) gene; these genes are known to be expressed in wood-forming tissues (Kirst et al. 2001). PCR amplification was performed using *Taq *DNA polymerase (Roche Diagnostics) at 55°C. Aliquots (5 μl) were removed after 20, 25, and 35 PCR cycles and assayed by agarose gel electrophoresis. cDNA synthesized was then used for cDNA-AFLP analysis and cDNA library construction.

### cDNA-AFLP analysis and library construction

cDNA-AFLP analysis was performed on the seven individual trees as described by Vos *et al*. with minor modifications [49]. One hundred nanogram of double-stranded cDNA was used as initial template for restriction digestion with *Pst*I and *Mse*I (KeyGene). For pre-amplification an *Mse*I primer and a *Pst*I primer without a selective nucleotide were combined. The amplification mixtures obtained were diluted 20-fold and 5 μl were used for the selective amplifications. Twelve *Mse*I primers with two or three selective nucleotides at the 3' end were combined with six *Pst*I primers with two or three selective nucleotides at the 3' end were used for the cDNA-AFLP analysis. One primer combination (*Mse*3 and *Pst*4) was selected for further studies as it had the highest polymorphisms and large numbers of scorable bands. The adaptors and primers used for cDNA-AFLP analysis can be viewed in additional file 4. The cDNA-AFLP fragments obtained by selective amplification were inserted into a pGEM T-easy vector system II cloning kit (Promega, Madison Visconsin) following the manufacturer's instructions. Cloned cDNA-AFLP fragments were then amplified with primers T7 and SP6 (Promega, Madison Visconsin) for arraying onto the microarray slide.

### cDNA library construction

The seven individual *E. grandis *trees were used to construct the cDNA library. The cDNA library was prepared by using the pGEM T-easy vector system II following the manufacture's instructions (Promega, Madison Visconsin). cDNA fragments were prepared by restriction-enzyme digestion of cDNA followed by ligation and transformation into *Escherichia coli *DH10α host cells. Individual colonies were plated on a grid followed by vector specific PCR using T7 and SP6 primers to verify that only single fragments were ligated. The cDNA library was stored at -80°C in 96 well microtiter plates in 75 μl of Luria Broth and 75 μl of a 50% glycerol solution. Before arraying, the individual clones were amplified using primers T7 and SP6 (Promega, Madison Visconsin) following the manufacturer's instructions (Promega, Madison Visconsin).

### Construction of the combined cDNA array

The 1578 cDNA clones used for microarray construction were a combination of two separate libraries, namely 810 cDNA library clones and 768 cDNA-AFLP cloned fragments. Amplified cDNA and cDNA-AFLP clones were purified using Multiscreen^® ^PCR Purification Plates (Millipore, Molsheim, France) and visualized on a 1% agarose Electro-Fast^® ^Stretch gel (ABgene, Epsom, UK). Purified clones were robotically printed onto silanised glass slides (Amersham Biosciences, Little Chalfont, UK) using an Array Spotter Generation III (Molecular Dynamics, Sunnyvale, CA, USA). The GUS and bar genes and a fungal rDNA internal transcribed spacer (ITS) fragment were also printed to serve as controls for global normalization. Fragment were arrayed in duplicate on each slide at 250-μM. A fungal rDNA internal transcribed spacer (ITS) fragment, water and a *bar *gene at concentrations of 50 ng/μ, 100 ng/μl, 150 ng/μl and 200 ng/μl were also printed to serve as controls.

### Hybridization of array slides

Seven *E. grandis *trees were used for microarray hybridizations. Probe cDNA from individual trees was prepared by restriction-enzyme digestion of cDNA (200 ng per tree) followed by ligation of restriction fragments to adapters and subsequent amplification following the protocol described above. Amplification products were column-purified using the QIAGEN PCR Purification Kit (QIAGEN, Valencia, CA) according to manufacturer's instructions. Probe cDNA labelling and hybridization were carried out following the procedure as described by Lezar *et al*. [50]. Reactions were spiked with cyanin-labeled controls for GUS, ITS and bar genes. Slides were scanned with a Genepix™ 4000B scanner (Axon Instruments, Foster City, CA, USA). The mean pixel intensity of each array that resulted from the individual hybridizations was quantified with the Array Vision 6.0 software (Imaging Research Inc., Molecular Dynamics, USA). For each hybridization experiment, one technical replicate (using independent labelling reactions) was performed, each replication consisting of a reverse labelling experiment. In addition, the whole experiment was repeated with one biological replicate labeled with Cy5 dye (i.e. three microarray slides were used in total for each sample).

### Image acquisition, data processing and statistical analysis

For each spot on the array, local background signal intensities were subtracted and signal intensities of duplicate spots on glass slides were averaged. A clone was considered to have hybridized to the array, if its fluorescence was more than two standard deviations above local background. Abnormal spots (e.g. high background, dust, irregularities) were manually flagged for removal. Anomalous spots detected through manual inspection were removed, if the signal intensity of an array feature varied more than 10% from the duplicate spot. Signal intensities of duplicate spots were then averaged and spots with a signal-to-noise ratio of less than two were rejected. Intensity values were normalized across slides by global regression on the spot intensity data for tree 1/23/4-HS, which was used as a reference for normalization of all spot intensity data (reference design). The control genes GUS, ITS (200 ng/μl, 100 ng/μl and 50 ng/μl) and bar genes printed in duplicate on the array served as a separate control to confirm that data across slides was normalized correctly. The statistical significance of changes in transcript abundance was estimated using the methods described by Wolfinger *et al*. [51]. Only genes with an average fold-change of 1.4 for biological replicates and a p value of 0.01 were considered to be differentially expressed.

For the cDNA-AFLP fragments, normalized signal intensity values were used to identify polymorphic fragments based on their bimodal distribution of their intensity values across slides as described by Lezar *et al*. [50]. Polymorphic markers identified were then scored for the absence (0) or presence (1) of the fragment in each of the respective *E. grandis *trees. The absence and presence of polymorphic spots were used for cluster analysis of the pairwise genetic distances between the hybridization profiles of individual *E. grandis *trees, using Spearman correlation and hierarchical clustering (CLUSTER, available at ). The clustering results were visualized with TreeView [27]. Gene expression patterns were identified by converting normalized data into log_2 _intensity values. Cluster analysis was performed on the normalized and mean-centered signal intensities using Pearson's correlation in the Cluster program and visualized in TreeView [27] in order to identify groups with similar expression patterns across the different *E. grandis *trees.

The data discussed above has been deposited at NCBI Gene Expression Omnibus (GEO) [52] and is accessible through GEO series accession number GSE14707.

### Data quality

To assess the reproducibility of the methods used in this study, biological and technical replicates from pools of xylem RNA samples were hybridized onto the slides each carrying the clones in duplicate. Approximately 16 of the 1578 background-corrected spots, representing about 1.0% of the cDNA present on the glass slide had signal intensities that varied more than 10% of the mean of the two replicates and were manually removed from subsequent data analysis. Spots excluded from analysis showed inaccuracies in signal intensities. This can be ascribed to variability in the experimental process introducing inaccuracies in labelling, array hybridisation, signal detection and quantification. This approach allowed us to obtain correct and repeatable scores, reducing the occurrence of spots that varied sufficiently to be erroneously classified.

### Sequencing and sequence analysis

Following microarray analysis, fragments of interest were re-amplified from the libraries using SP6 and T7 primers. Amplification products were column-purified using the QIAGEN PCR Purification Kit (QIAGEN, CA) according to manufacturer's instructions. Sequencing reactions were carried out at Inqaba (South Africa). Single-pass partial sequences were obtained with universal T7 primer. After manual removal of ambiguous sequences, sequences were assigned putative identities by translating BLAST (BLASTX) [53], against the non-redundant protein database of the National Centre for Biotechnological Information database [54]. E-values were considered significant if they were below 10^-3^.

### Confirmation of expression profiles by qRT-PCR

A subset of five genes was used to verify the microarray results. The five fragments that were chosen represent varying expression profiles across the *E. grandis *trees. Primer pairs were designed to UDP-glucuronic acid decarboxylase 3, hpt2 gene, D-isomer specific 2-hydroxyacid dehydrogenase, partial cDNA sequence of caffeoyl-CoA 3-O-methyltransferase (CCoAOMT) and protein-L-isoaspartate O-methyltransferase. Microarray analysis suggested that ribosomal RNA was expressed constitutively and was, therefore, used as a reference. The relative transcript abundance was detected by a Light Cycler (Roche Diagnostics, Basel, Switzerland) and Light Cycler FastStart DNA Master^PLUS ^SYBR Green I kit (Roche Diagnostics). PCR reactions were performed in a total volume of 20 μl containing 5 ng of single-stranded cDNA, 1 × Light Cycler FastStart DNA Master^PLUS ^SYBR Green I Master Mix and 1 μM of each primer. A negative control was run without cDNA template with every assay to assess the overall amplification specificity. Relative quantification was performed using the LightCycler software version 3.5.3 (Roche).

## Abbreviations

cDNA: clonal deoxyribonucleic acid; cDNA-AFLP: clonal deoxyribonucleic acid-amplified fragment length polymorphism; EST: expressed sequence tag; mRNA: messenger ribonucleic acid; PCR: polymerase chain reaction; qRT-PCR: quantitative reverse transcription polymerase chain reaction.

## Authors' contributions

EB: conceived the study, participated in its design and co-ordination and drafted the manuscript. CAVS: preparation of biological samples, construction of libraries for arraying. SL: microarray study, qRT-PCR analysis, statistical analysis, helped to draft the manuscript. All authors read and approved the final paper.

## Supplementary Material

Additional file 1 **Functional chart of microarray results**. Functional classification of 80 expression patterns from the cDNA and cDNA-AFLP arrays. ESTs with BLAST E-values < 10^-3 ^were classified into MIPS functional categories according to stress, signal transduction, cell biogenesis, cell growth, protein metabolism, transcription, energy, metabolism and unclear classification. The no hits category corresponded to proteins that had no significant sequence similarity to the existing databases. Click here for file

Additional file 2 **Hierarchical clustering of binary scores of seven *E. grandis *trees**. TreeView (Eisen et al., 1998) representation of clustering of 768 hybridization profiles of seven *E. grandis *trees based on cDNA xylem library microarray analysis. Columns represent hybridization profiles of the individuals and rows represent the binary scores. Green bars indicate absence (-1) and red bars indicate presence (1) of an array feature, respectively, and black bars indicate an intermediate values of zero. The numbers next to the rows indicate the spot number in the array. Box 1 represent areas that are highly expressed in the two high splitters and box 2 represent areas of high and low expression in the two low splitters. Click here for file

Additional file 3 ***E. grandis *trees used for the RNA isolation**. Table displaying *E. grandis *trees used for the RNA isolation. Click here for file

Additional file 4 **Adaptors and primers used for cDNA-AFLP analysis**. Table displaying adaptors and primers used for cDNA-AFLP analysis. Click here for file

## References

[B1] Godsmark R (2008). The South African Forestry and Forest Products Industry 2007. Forestry S A.

[B2] Malan FS (1979). The control and-splitting in saw logs: A short literature review. South African Forestry Journal.

[B3] Barros E, Verryn S, Hettasch M (2002). Identification of PCR-based markers linked to wood splitting in *Eucalyptus grandis*. Ann For Sci.

[B4] Fukuda H (2004). Signals that control plant vascular cell differentiation. Nat Rev Mol Cell Biol.

[B5] Demura T, Fukuda H (2007). Transcriptional regulation in wood formation. Trends in Plant Science.

[B6] Durrant WE, Rowland O, Piedras P, Hammond-Kosack KE, Jones JDG (2000). cDNA-AFLP reveals a striking overlap in the race-specific resistance and wound response expression profiles. Plant Cell.

[B7] Jones CS, Davies HV, Taylor MA (2000). Profiling of changes in gene expression during raspberry (*Rubus idaeus*) fruit ripening by application of RNA fingerprinting techniques. Planta.

[B8] Ranik M, Creux NM, Myburg AA (2006). Within-tree transcriptome profiling in wood-forming tissues of a fast-growing *Eucalyptus tree*. Tree Physiol.

[B9] Allona I, Quinn M, Shoop E, Swope K, St Cyr S, Carlis J, Riedl J, Retzel E, Campbell MM, Sederoff R, Whetten RW (1998). Analysis of xylem formation in pine by cDNA sequencing. Proc Natl Acad Sci USA.

[B10] Pavy N (2005). Large-scale statistical analysis of secondary xylem ESTs in pine. Plant Mol Biol.

[B11] Ramussen-Poblete S, Valdes J, Gamboa MC, Valenzuela PDT, Krauskopf E (2008). Generation and analysis of an Eucalyptus globulus cDNA library constructed from seedlings subjected to low tempreture conditions. E J Biotech.

[B12] Oh S, Park S, Han KH (2003). Transcriptional regulation of secondary growth in Arabidopsis thaliana. J Exp Bot.

[B13] Cánovas F, Dumas-Gaudot E, Recorbet E, Jorrin J, Mock H-P, Rossignol M (2004). Plant proteome analysis. Proteomics.

[B14] Kirst M, Myburg AA, De Leon JP, Kirst ME, Scott J, Sederoff R (2004). Coordinated genetic regulation of growth and lignin revealed by quantitative trait locus analysis of cDNA microarray data in an interspecific backcross of Eucalyptus. Plant Phys.

[B15] Andersson-Gunnerås S, Mellerowicz EJ, Love J, Segerman B, Ohmiya Y, Coutinho PM, Nilsson P, Henrissat B, Moritz T, Sundberg B (2006). Biosynthesis of cellulose-enriched tension wood in *Populus*: global analysis of transcripts and metabolites identifies biochemical and developmental regulators in secondary wall biosynthesis. Plant J.

[B16] Qui D, Wilson IW, Gan S, Washusen R, Moran GF, Southerton SG (2008). Gene expression in Eucalyptus branch wood with marked variation in cellulose microbibril orientation and lacking G-layers. New Phytol.

[B17] Yang SH, van Zyl L, No EG, Loopstra CA (2004). Microarray analysis of genes preferentially expressed in differentiating xylem of loblolly pine (Pinus taeda). Plant Science.

[B18] Heller G, Adomas A, Li G, Osborne J, van Zyl L, Sederoff R, Finlay RD, Stenlid J, Asiegbu FO (2008). Transcriptional analysis of *Pinus sylvestris *roots challenged with the ectomycorrhizal fungus *Laccaria bicolor*. BMC Plant Biology.

[B19] Schrader J, Nilsson J, Mellerowicz E, Berglund A, Nilsson P, Hertzberg M, Sandberg G (2004). A high-resolution transcript profile across the wood-forming meristem of poplar identifies potential regulators of cambial stem cell identify. The Plant Cell.

[B20] Israelsson M, Eriksson ME, Hertzberg M, Aspeborg H, Nilsson P, Moritz T (2003). Changes in gene expression in the wood-forming tissue of transgenic hybrid aspen with increased secondary growth. Plant Mol Biol.

[B21] Demura T, Tashiro G, Horiguchi G, Kishimoto N, Kubo N, Matsuoka N, Minami A, Nagat-Hiwatashi M, Nakamura K, Okamura Y, Sassa N, Suzuki S, Yazaki J, Kikuchi S, Fukuda H (2002). Visualization by comprehensive microarray analysis of gene expression programs during transdifferentiation of mesophyll cells into xylem cells. Proc Natl Acad Sci USA.

[B22] Foucart C, Paux E, Ladouce N, San-Clemente H, Grima-Pettenati J, Sivadon P (2006). Transcript profiling of a xylem vs phloem cDNA subtractive library identifies new genes expressed during xylogenesis in *Eucalyptus*. New Phytol.

[B23] Wittenberg AHJ, Lee T van der, Cayla C, Kilian A, Visser RGF, Schouten HJ (2005). Validation of the high-throughput marker technology DArT using the model plant *Arabidopsis thaliana*. Mol Gen Genomics.

[B24] Akbari M, Wenzl P, Caig V, Carling J, Xia L, Yang S, Uszynski G, Mohler V, Lehmensiek A, Kuchel H, Hayden MJ, Howes N, Sharp P, Vaughan P, Rathmell B, Huttner E, Kilian A (2006). Diversity arrays technology (DArT) for high-throughput profiling of the hexaploid wheat genome. Theor Appl Genet.

[B25] Wenzl P, Li H, Carling J, Zhou M, Raman H, Paul E, Hearnden P, Maier C, Xia L, Caig V, Ovesná J, Cakir M, Poulsen D, Wang J, Raman R, Smith KP, Muehlbauer GJ, Chalmers KJ, Kleinhofs A, Huttner E, Kilian A (2006). A high-density consensus map of barley linking DArT markers to SSR, RFLP and STS loci and agricultural traits. BMC Genomics.

[B26] Wenzl P, Raman H, Wang J, Zhou M, Huttner E, Kilian A (2007). A DArT platform for quantitative bulked segregant analysis. BMC Genomics.

[B27] Eisen MB, Spellman PT, Brown PO, Botstein D (1998). Cluster analysis and display of genome-wide expression patterns. Proc Natl Acad Sci USA.

[B28] Wolfinger RD, Gibson G, Wolfinger ED, Bennett L, Hamadeh H, Bushel P, Afshari C, Paules RS (2001). Assessing gene significance from cDNA microarray expression data via mixed models. J Comput Biol.

[B29] Schoof H, Zaccaria P, Grundlach H, Lemcke K, Rudd S, Kolesov G, Arnold R, Mewes HW, Mayer KF (2002). MIPS Arabidopsis thaliana Database (MAtDB): an integrated biological knowledge resource based on the first complete plant genome. Nucl Acids Res.

[B30] Iliev EA, Xu W, Polisensky DH, Oh M-H, Torisky RS, Clouse SD, Braam J (2002). Transcriptional and posttranscriptional regulation of Arabidopsis *TCH4 *expression by diverse stimuli. Roles of cis regions and brassinosteroids. Plant Physiol.

[B31] Pilate G, Dejardin A, Laurans F, Leple JC (2004). Tension wood as a model for functional genomics of wood formation. New Phytol.

[B32] Kanter U, Usadel B, Guerineau F, Li Y, Pauly M, Tenhaken R (2005). The inositol oxygenase gene family of Arabidopsis is involved in the biosynthesis of nucleotide sugar precursors for cell-wall matrix polysaccharides. Planta.

[B33] Bindschedler LV, Tuerck J, Maunders M, Ruel K, Petit-Conil M, Danoun S, Boudet A-M, Joseleau J-P, Bolwell GP (2007). Modification of hemicellulose content by antisense down-regulation of UDP-glucuronate decarboxylase in tobacco and its consequences for cellulose extractability. Phytochemistry.

[B34] Xu G, Lehmann R, Schleicher E, Haring HU, Liebich H (1998). Advantages in the analysis of UDP-sugars by capillary electrophoresis-comparison of the conventional HPLC method with two new capillary electrophoretic micro-procedures. Biomed Chromat.

[B35] Bourquin V, Nishikubo N, Abe H, Brumer H, Denman S, Eklund M, Christiernin M, Teeri TT, Sundberg B, Mellerowicz EJ (2002). Xyloglucan endotransglycosylases have a function during the formation of secondary cell walls of vascular tissues. Plant Cell.

[B36] Nishikubo N, Awano T, Banasiak A, Bourquin V, Ibatullin F, Funada R, Brumer H, Teeri TT, Hayashi T, Sundberg B, Mellerowicz EJ (2007). Xyloglucan *Endo*-transglycosylase (XET) Functions in Gelatinous Layers of Tension Wood Fibers in Poplar – A Glimpse into the Mechanism of the Balancing Act of Trees. Plant and Cell Physiol.

[B37] Trugilho PF, Lima JT, Rosado SCS (2005). Assessment of growth stresses in Eucalyptus trees and their relationship with the characteristics of growth and some wood properties. International Conference on Plantation Eucalyptus: Challenge in product development: 2005; ZhanJian – China.

[B38] Chabannes M, Barakate A, Lapierre C, Marita JM, Ralph J, Pean M, Danoun S, Halpin C, Grima-Pettenati J, Boudet AM (2001). Strong decrease in lignin content without significance alteration of plant development is induced by simultaneous down-regulation of cinnamoyl CoA reductase (CCR) and cinnamyl alcohol dehydrogenase (CAD) in tobacco plants. Plant Journal.

[B39] Martz F, Maury S, Pincon G, Legrand M (1998). cDNA cloning, substrate specificity and expression study of tobacco caffeoyl-CoA 3-*O*-methyltransferase, a lignin biosynthetic enzyme. Plant Mol Biol.

[B40] Zhong RQ, Morrison WH, Himmelsbach DS, Poole FL, Ye ZH (2000). Essential role of caffeoyl coenzyme A *O*-methyltransferase in lignin biosynthesis in woody poplar plants. Plant Physiol.

[B41] Paux E, Carocha V, Marques C, de Sousa AM, Borralho N, Sivadon P, Grima-Pettenati J (2005). Transcript profiling of Eucalyptus xylem genes during tension wood formation. New Phytol.

[B42] Seifert GJ, Roberts K (2007). The biology of arabinogalactan proteins. Ann Rev of Plant Biol.

[B43] Paux E, Tamasloukht M, Ladouce N, Sivadon P, Grima-Pettenati J (2004). Identification of genes preferentially expressed during wood formation in *Eucalyptus*. Plant Mol Biol.

[B44] Sterky F, Regan S, Karlsson J, Hertzberg M, Rohde A, Holmberg A, Amini B, Bhalerao R, Larsson M, Villarroel R, Van Montagu M, Sandberg G, Olsson O, Teeri OO, Boerjan W, Gustafsson P, Uhlen M, Sundberg B, Lundeberg J (1998). Gene discovery in the wood-forming tissues of poplar: Analysis of 5,692 expressed sequence tags. Proc Natl Acad Sci USA.

[B45] Kirst M (2003). Apparent homology of expressed genes from wood-forming tissues of loblolly pine (*Pinus taeda *L.) with Arabidopsis thaliana. Proc Natl Acad Sci USA.

[B46] Sterky F, Bhalerao RR, Unneberg P, Segerman B, Nilsson P, Brunner AM, Charbonnel-Campaa L, Lindvall JJ, Tandre K, Strauss SH, Sundberg B, Gustafsson P, Uhlen M, Bhalerao RP, Nilsson O, Sandberg G, Karlsson J, Lundeberg J, Jansson S (2004). A *Populus *EST resource for plant functional genomics. Proc Natl Acad Sci USA.

[B47] Turner P (2000). The prediction and selection of *E. grandis *solid wood: Phase one. CSIR report ENV-P-C 2000-019.

[B48] Chang S, Puryear J, Cairney J (1993). A simple and efficient method for isolating RNA from Pine trees. Plant Mol Biol Rep.

[B49] Vos P, Hogers R, Bleeker M, Reijans M, Lee T van de, Hornes M, Fritjers A, Pot J, Peleman J, Kuiper M, Zabeau M (1995). AFLP: a new technique for DNA fingerprinting. Nucleic Acids Res.

[B50] Lezar S, Myburg AA, Berger DK, Wingfield MJ, Wingfield BD (2004). Development and assessment of microarray-based DNA fingerprinting in *Eucalyptus grandis*. Theor Appl Genet.

[B51] Wolfinger RD, Gibson G, Wolfinger ED, Bennett L, Hamadeh H, Bushel P, Afshari C, Paules RS (2001). Assessing gene significance from cDNA microarray expression data via mixed models. J Comput Biol.

[B52] NCBI Gene Expression Omnibus (GEO). http://ncbi.nlm.nih.gov/geo/.

[B53] Altschul SF, Gish W, Miller W, Meyers EW, Lipman DJ (1990). Basic local alignment search tool. J Mol Biol.

[B54] National Centre for Biotechnological Information. http://www.ncbi.nlm.nih.gov.

