# Supplementary figures and images for: A microarray-based method for the parallel analysis of genotypes and expression profiles of wood-forming tissues in Eucalyptus grandis
Source: BMC Biotechnol. 2009 May 27;9:51. doi: 10.1186/1472-6750-9-51 (PMC2698882; doi:10.1186/1472-6750-9-51)

## Additional file 1

### *Broad functional classification of 80 ESTs*

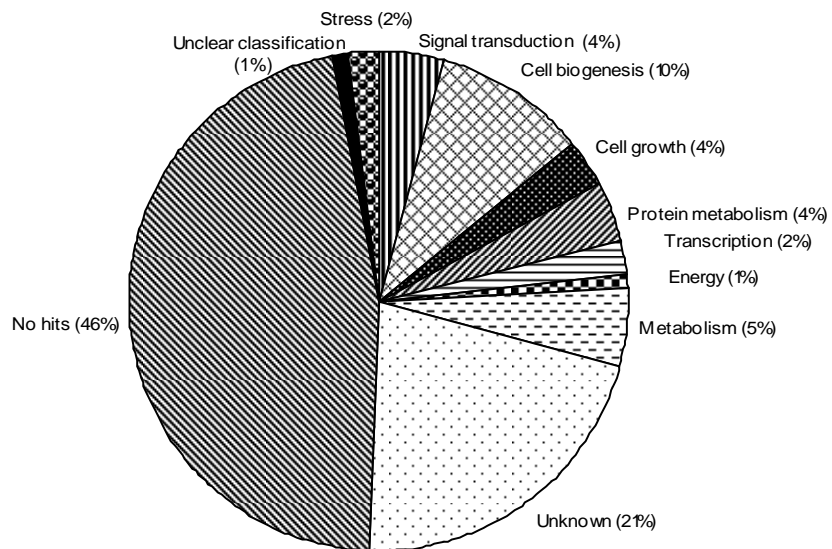

Supplement: Additional file 1 — Functional chart of microarray results. Functional classification of 80 expression patterns from the cDNA and cDNA-AFLP arrays. ESTs with BLAST E-values < 10-3 were classified into MIPS functional categories according to stress, signal transduction, cell biogenesis, cell growth, protein metabolism, transcription, energy, metabolism and unclear classification. The no hits category corresponded to proteins that had no significant sequence similarity to the existing databases. [file 1472-6750-9-51-S1.pdf]

**Additional file 2**  
*Hierarchical clustering of binary scores of seven E. grandis trees*

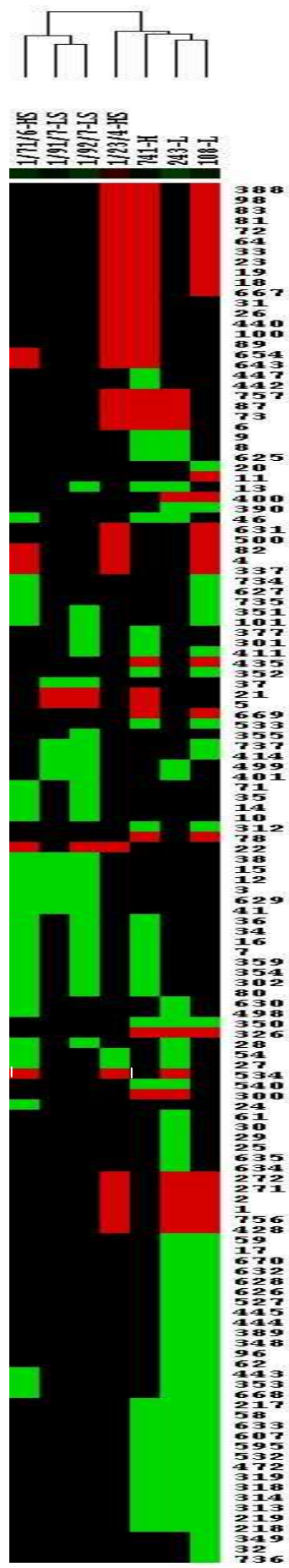

Supplement: Additional file 2 — Hierarchical clustering of binary scores of seven E. grandis trees. TreeView (Eisen et al., 1998) representation of clustering of 768 hybridization profiles of seven E. grandis trees based on cDNA xylem library microarray analysis. Columns represent hybridization profiles of the individuals and rows represent the binary scores. Green bars indicate absence (-1) and red bars indicate presence (1) of an array feature, respectively, and black bars indicate an intermediate values of zero. The numbers next to the rows indicate the spot number in the array. Box 1 represent areas that are highly expressed in the two high splitters and box 2 represent areas of high and low expression in the two low splitters. [file 1472-6750-9-51-S2.pdf]
